# Supplementary material for: Managing the Consequences of Oncological Major Surgery: A Short- and Medium-Term Skills Assessment Proposal for Patient and Caregiver through M.A.D.I.T. Methodology
Source: Behav Sci (Basel). 2022 Mar 15;12(3):77. doi: 10.3390/bs12030077 (PMC8945744; doi:10.3390/bs12030077)
Supplement: Supplementary file 1 [file behavsci-12-00077-s001.zip › behavsci-1592655-supplementary.pdf]

Table S1. Exemplification of the different levels of competence observed through the excerpts of text collected during the interviews.

COMPETENCE	*EXAMPLE OF QUESTION*	*SAMPLE RESPONSE TEXT COLLECTED*	*FALLOUT DESCRIPTION*	*CRITERIA*
Preview of future scenarios	*We ask you to imagine that you are at the check-up about 3 months after the surgery: when the surgeon will ask you to describe your physical condition after the surgery, what can be your answers?*	HIGH Competence*:*<br>*From what the doctors have told me, I should be able to answer "fine," but you have to wait for the results of the histological exam as well.*	In narrating what he anticipates may happen, the patient uses third-party references (not his personal theories) and shareable ones, thus making his anticipation more accurate and putting him in a position to manage what might happen.	The patient can depict multiple scenarios which can occur, using shared criteria.
		MEDIUM Competence*:*<br>*I think, if it's like now, it's fine, actually not fine, great, if it's like now. I introduce eating a little bit at a time, we need to stay grounded.*	The patient makes reference to the future, but not using third party and shareable elements, the anticipation could be based even only on sensations and thoughts, making it less precise and opening to unforeseen events for which there has been no preparation.	The patient can depict multiple scenarios which can occur, but uses only personal criteria and opinions.
		LOW competence*:*<br>*In three months, I will answer him on the base of what I'll feel, I can't answer now for tomorrow.*	By not thinking about what might happen in the future, the patient is not in a position to develop strategies for managing potential critical aspects of the hospital stay.	The patient depicts just one possible scenario, using personal criteria and opinions
Situation evaluation	*Once out of the hospital, how will you deal with the period after discharge?*	HIGH Competence*:*<br>*I only have my husband at home, I will try to put in all the good will, managing as best I can, seeking help from the doctor for when I have pain, otherwise cooperating with my husband and trying to do things normally.*	The patient takes into consideration situational elements in order to offer evaluations regarding how he will move, thus highlighting that he is in a position to make different evaluations when the current situation changes.	Context elements about present condition are used to manage evaluations or to describe the situation

		MEDIUM Competence*:*<br>*Having a mindful standard of living and not overdoing it in nutrition.*	The patient considers the elements of the situation, but does not use them to offer evaluation.	Context elements are only mentioned, but not used to performs any sort of evaluation
		LOW competence*:*<br>*I will need to adjust the situation.*	The patient does not refer to the specific situational elements, opening up the possibility of inaccurate assessments being made that may impact the health care situation.	Context elements are either not mentioned or addressed using opinions and personal beliefs
Preview repercussion of the own actions	*We ask you to imagine that you are 3 months from now in a situation where you have to choose whether or not to do a certain thing. However, you are not sure whether this is in line with the instructions given to you by the medical staff. What would you consider when deciding what to do?*	HIGH Competence*:*<br>*If I'm not sure it's on the line, first thing is to ask the medical staff. If they tell me no, I would act also based on what they say. It's not like I want to risk it at all.*	The patient in anticipating how he or she would move presents a scenario as a possibility along with the third-party, agreeable elements he or she has considered; this makes the anticipation more accurate and therefore manageable before it happens.	The patient can depict multiple scenarios which can occur, using shared criteria, about implications of his/her actions
		MEDIUM Competence*:*<br>*I would try to do, because I underestimated this situation, I had a heavy breakdown.*	The patient presents a scenario in terms of possibilities but does not make explicit the criteria he/she considered: this opens up either the possibility that the references used are third parties and shared, making the anticipation accurate, or that the criteria are the patient's specific thoughts and hopes.	The patient can depict multiple scenarios which can occur, but uses only personal criteria and opinions to consider implications of his/her actions

		LOW Competence:<br>*I don't do it.*	The patient imagines only one possible scenario and does not make explicit according to what criteria. This makes the anticipation more imprecise and therefore does not put the patient in a position to manage sooner what could critically happen.	The patient depicts just one possible scenario, using personal criteria and opinions, about implications of his/her actions
Use of resources	*After your discharge from the hospital for the surgery you underwent: who are the people who could help you? In what way?*	HIGH Competence:<br>*Family members especially, for food preparation, as it will change my nutrition.*	The patient considers the resources (in terms of people close to him/her in this case), which he/she has at his disposal to manage the consequences of the intervention, and he/she is able to make explicit how they might help him. This makes it possible to anticipate the effective use of these resources, which allows for effective post-operative management.	Patient explicits the value of the context elements as resources, using explicit criteria
		MEDIUM Competence:<br>*Family members and physician, oncologist.*	The patient simply mentions the resources he or she would have available, without highlighting how they might be useful, reducing the likelihood that they will actually be used.	Context elements are considered resources by the patient, without explicating criteria
		LOW Competence:<br>*Me and me. I would love to drink wine and eat, I've never held myself.*	The patient does not consider resources outside of themselves that might be helpful to them in managing postoperatively.	Patient does not refer to the context elements as possible resources

Table S2. Patient sample characteristics and frequency

**Sex**		**Cancer Localization**	
Male	30	Cardias	6
Female	17	Esophagus	18
**Age (years)**		Stomach	14
31-50	3	Colon	4
51-65	18	Rectum	5
66-75	17	**Neoadjuvant Therapy**	
76-90	9	Neoadjuvant Chemotherapy	14
**Marital status**		Neoadjuvant Chemo-radiotherapy	15
Married	26	**Type of surgery**	
Unmarried	5	Intrathoracic Esophagogastroplasty	17
Divorced	4	Cervical Esophagogastroplasty	4
Missing data	12	Esophagectomy	3
**Children**		Gastrectomy	7
Yes	24	Gastro-resection	6
No	8	Abdominal-perineal amputation	1
Missing data	15	Sigma resection	2

**Level of education**		Anterior rectal resection	3
Primary School	12	Colic resection	4
Middle School	12	**General comorbidities**	
High School	8	Yes	36
Degree	9	No	11
Missing data	6		
**Other neoplasms**			
Yes	4	No	43
**Employment**		**Cardiovascular diseases**	
Employed	18	Yes	18
Not Employed	29	No	29
**Psychiatric comorbidities**		**Chronic diseases**	
Yes	4	Yes	31
No	43	No	16
**Previous surgery**			
Yes	20	No	27
